# Concerns of Using Large Language Models in Health Care Research and Practice: Umbrella Review

**DOI:** 10.2196/87804

**Published:** 2026-05-15

**Authors:** Feyza Yarar, Pauline Addis, Megan Fairweather, Dawn Craig, Hannah O'Keefe

**Affiliations:** 1 Population Health Sciences Institute, Faculty of Medical Sciences, Newcastle University, Framlington Place, Newcastle-Upon-Tyne, England, NE2 4HH, United Kingdom, 44 7826034122; 2 NIHR (National Institute of Health and Care Research) Innovation Observatory, Newcastle University, Newcastle-Upon-Tyne, England, United Kingdom

**Keywords:** artificial intelligence, umbrella review, concerns, health and social care, life sciences

## Abstract

**Background:**

Large language models (LLMs), such as ChatGPT (OpenAI), are rapidly evolving, and their applications in health care are increasing. There is a growing demand for automation of routine tasks and a drive to use LLMs or similar to support research.

**Objective:**

This umbrella review examines concerns of health care professionals and researchers related to the use of LLMs in health care research and practice. We aimed to identify common issues raised and the implications for patient care, policy, and practice.

**Methods:**

A protocol was registered on PROSPERO (CRD420250640997). Searches were conducted in 7 databases (Ovid MEDLINE, Ovid Embase, Scopus, Web of Science, JBI Database of Systematic Reviews and Implementation Reports, Cochrane Database of Systematic Reviews, and Epistemonikos) in February 2025 and updated in February 2026. Screening was conducted in 2 stages, with independent screening by 2 reviewers. Studies published in the English language after January 2017 with at least one outcome expressing concerns of LLM or generative artificial intelligence use in health care research were included. The included studies were quality appraised for risk of bias and certainty of the evidence using AMSTAR-2 (A Measurement Tool to Assess Systematic Reviews) and GRADE (Grading of Recommendations Assessment, Development, and Evaluation), respectively. Data was extracted using a piloted form and narratively synthesized following SWiM guidelines and the PRIOR (Preferred Reporting Items for Overviews of Reviews) checklist.

**Results:**

The search retrieved 448 systematic reviews, of which 42 met the inclusion criteria. Further, 12 distinct populations were identified, including researchers and clinicians in various medical specialties. The included reviews were assessed to be of very poor quality, and the level of overlap between primary studies could not be determined. Additionally, 15 reviews focused on ChatGPT, a further 15 on two or more LLMs, and 12 on generic artificial intelligence. Thus, 3 main themes emerged from the narrative synthesis. In order of most to least frequently discussed: (1) technical capability; (2) ethical, legal, and societal; and (3) costs.

**Conclusions:**

To our knowledge, this is the first umbrella review to address the concerns of LLMs in health care research and practice. Thematic analyses provided insight into the complexity of different perspectives, and by using a whole population approach, it demonstrates common narratives. However, the poor quality of the included studies and potential overlap of results are substantial limitations. Data quality is at the heart of these concerns, and combative action must ensure health care professionals and researchers have the resources required to overcome these apprehensions. Ethical, legal, and societal implications of artificial intelligence use were also commonly raised. As technology accelerates and demands on health care increase, we must adapt and embrace change with equity, diversity, inclusion, and safety at the core.

## Introduction

### Background

Currently, we live in an era where an abundance of data is being produced worldwide. While the term “big data” is generally used for predictive analytics, health care data can be considered “big data” by definition, as it is high in volume, velocity, variety, and veracity [1]. Big data is more suited to computational analysis, rather than traditional manual methods, and automating this analysis is an attractive proposition. The growing need to handle large datasets in the field of health care has led researchers to seek to leverage artificial intelligence (AI) as a means of automation [2]. The recent development of generative artificial intelligence (GenAI), particularly large language models (LLMs), has opened new frontiers in data handling [3]. In brief, GenAI uses advanced architectures, model context, and user prompts to recognize patterns in extensive data sets and generate original outputs. In the case of LLMs, this is done via transformer architectures, advanced neural networks designed to deliver next-token prediction [4].

LLMs are a versatile tool with the potential to transform health care research, but they also pose distinct challenges. As with other health care innovations, the risks and benefits of using LLMs should be weighed before implementation. Increasingly, there is a growing effort to develop strategies for the responsible use of AI. For example, leading journals do not accept papers with AI as an author, and NICE (National Institute of Health and Care Excellence) has guidelines on the use of AI in evidence generation [5,6]. Internationally, the European Union is expected to create the first AI law to be enforced in 2026, stratifying AI systems by risk level and regulating them accordingly [7]. Canada has also drafted legislation on AI [8]. Most laws focus on AI companies rather than individuals and have not yet taken effect. In addition to specific laws surrounding AI, it is crucial to comply with current unrelated but relevant laws, such as the General Data Protection Regulation (GDPR), in the interest of safety [9,10]. This will involve enhancing data security and clearly defining accountability [11,12].

The use of health care data is already bound by GDPR, and LLMs have been used in health care research applications ranging from the analysis of medical records and images to enhancing drug discovery and informing the formulation of new treatments [13-17]. Automated documentation could further assist clinical practice through use cases such as writing discharge summaries and personalized treatment or medication management plans [18,19]. This is particularly timely in the United Kingdom, where the government has pledged to embed AI throughout the National Health Service to support routine administration tasks [20]. There has also been a push toward automation methodologies in health and care research to deliver timely insights. This is particularly true in the field of evidence synthesis. However, the conversation and movement toward automation in this field have been ongoing for 2 decades with little to no progress [21-23].

As the speed of technological improvements accelerates, this can often outpace our ability to understand, assess, and mitigate concerns regarding AI [24]. Such concerns include reliability, accuracy, transparency, various ethical, security, and privacy concerns, as well as environmental concerns [25-27]. Such issues may be intrinsic to the LLM, reflecting its technical capabilities; extrinsic to the LLM, often relating to how it is used; or they may fall under both categories. When considering health care data, protection, security, and accuracy are paramount. As such, it is crucial to understand the views of individuals working in health care practice and research surrounding the use of LLMs. While research has been conducted to understand different population views, there has been no effort to cross-reference and triangulate these views. This is imperative to understand the landscape as a whole and promote multidisciplinary combative action. Thus, this umbrella review examines the concerns of health care professionals and researchers to identify areas for improvement and understand the implications for practice. It is anticipated that through the robust identification of issues, steps can be taken to mitigate concerns, instill confidence in users of AI, and that the use of AI will become more responsible.

### Aims and Objectives

We aimed to map the concerns associated with the use of LLMs in health care research and practice through the following objectives: (1) identify systematic reviews that report concerns of health care professionals and health care researchers, and (2) perform qualitative analysis of the findings using inductive and deductive thematic analysis.

## Methods

### Study Design

Following a scoping search that confirmed the feasibility of this study, a protocol for the systematic review was developed and registered with PROSPERO (CRD420250640997) on February 24, 2025. No amendments were made to the information provided in the protocol. The umbrella review was conducted in accordance with the PRISMA (Preferred Reporting Items for Systematic Reviews and Meta-Analyses) guidelines and the synthesis without meta-analysis guidelines, and reported using the PRIOR (Preferred Reporting Items for Overviews of Reviews) checklist, PRISMA-S (Preferred Reporting Items for Systematic Reviews and Meta-Analyses - Search), and PRISMA 2020 Abstract checklist (Checklists 1-3) [28-31].

### Eligibility Criteria

The SPIDER (Sample, Phenomenon of Interest, Design, Evaluation, Research Type) framework was used to outline the inclusion criteria as follows:

- Sample: health care professionals and researchers.
- Phenomenon of interest: LLMs or GenAI.
- Design: systematic reviews.
- Evaluation measures: reporting of concerns.
- Research type: qualitative research.

We included systematic reviews published since January 2017 (the year before the first LLMs being introduced publicly to ensure robust coverage of dates), where an outcome of the review was concerns surrounding the use of GenAIs or LLMs in a health and social care research context. We considered systematic reviews as defined by the authors, provided the methodology followed a recognizable process (ie, searching, screening, data extraction, risk of bias, and synthesis). Preprints and nonpeer-reviewed papers were excluded (Textbox 1).

Textbox 1. **Inclusion and exclusion criteria for the umbrella review applied to the retrieved search results.**

Inclusion criteria:

- Published since January 2017
- Generative artificial intelligence (GenAI) or large language models (LLMs) used in a health care practice and research context
- Outcome for concerns surrounding the use of GenAIs or LLMs
- Systematic reviews

Exclusion criteria:

- Published before January 2017
- Research context not related to health care practice and research
- GenAIs or LLMs concerns not listed as an outcome
- Primary studies
- Letters
- Editorials reviews
- Conference abstracts
- Commentaries

### Information Sources

Searches were completed on February 26, 2025, and updated on February 25, 2026, in seven databases: (1) Ovid MEDLINE (R) and Epub Ahead of Print, In-Process, In-Data-Review and Other Non-Indexed Citations, daily and versions; (2) Ovid Embase; (3) Scopus; (4) Web of Science; (5) JBI Database of Systematic Reviews and Implementation Reports; (6) Cochrane Database of Systematic Reviews; and (7) Epistemonikos.

Study registry searches, purposeful searching of gray literature sources, and citation chaining were neither performed nor were authors independently contacted for further data.

### Search Strategy

A de novo search strategy was developed in MEDLINE (Ovid) using the phenomenon of interest and evaluation measures concepts of the SPIDER framework: (ethic* or concern* or raises questions or equality or equity or racial or discriminat* or EDI or (equity diversity and inclusion) or adversely or perpetuat* or persist* or bolster or pitfall* or controvers* or worry or barrier or impede or obstacle or limitation or hindrance or hurdle) AND ((LLM or large language model or GenAI or generative AI or ChatGPT or OpenAI or gpt or Gemini or DeepSeek or LlaMA or Falcon or Cohere or PaLM or Claude v1 or autoregressive language or encoder-decoder or decoder or transformer or prompt engineer) AND (research or academ*). The strategy was peer reviewed by an information specialist and translated into other databases as appropriate (Multimedia Appendix 1). A date limit of 2017 onward was applied to all searches, and results were limited to systematic reviews using built-in functions in each database. No language restrictions were applied.

### Selection Process

The records from the databases were imported into Rayyan, a free online screening platform, and duplicates were removed [32]. The remaining systematic reviews were initially screened by title and abstract in duplicate (FY and PA or MF). Discrepancies were resolved by discussion, and HO provided a final judgment when a consensus could not be reached. Systematic reviews taken forward for full-text screening were independently screened by 2 reviewers (FY and PA or MF). Again, discrepancies were resolved by discussion.

### Data Collection Process

The data extraction template was initially trialed on 4 systematic reviews selected at random. Further, 2 reviewers independently extracted data from 50% of the included studies (FY and PA or MF). Discrepancies were resolved via discussion or consultation with HO. The remaining studies were extracted by 1 reviewer (FY) with discussion when required.

### Data Items

The following data were extracted: first author, year, title, DOI (Digital Object Identifier), journal, country of the first author, health care or research field, the LLM assessed, number of included studies, included study designs, inclusion criteria, exclusion criteria, key concerns, population raising the concern, notable quotes, statistical methods, and declared limitations.

### Risk of Bias Assessment

AMSTAR-2 (A Measurement Tool to Assess Systematic Reviews) was used to assess risk of bias, including reporting bias. The 16 questions outlined in AMSTAR-2 were applied independently by 2 reviewers to each included study, following the guidelines for each question or domain included [33,34].

### Synthesis Methods

The narrative synthesis was conducted by a single reviewer (FY), using a thematic analysis approach with inductive and deductive coding [35]. No statistical synthesis or meta-analysis was conducted, and quantitative effect measures were neither appropriate nor available given the qualitative nature of concerns. Concerns from the data extraction tables were analyzed and inductively synthesized into codes. Each systematic review was deductively analyzed to determine whether these codes were present in the text. The codes were organized and synthesized into main themes. The main themes were subsequently organized into overarching themes. An analysis by population was also conducted, which recorded the number of systematic reviews for a particular population that highlighted each of the coded concerns.

### Certainty Assessment

The application of GRADE (Grading of Recommendations Assessment, Development, and Evaluation) to systematic reviews of qualitative research gives a measure of how well the findings reflect the phenomenon of interest and provides an indicator of certainty around the evidence. GRADE was applied to assess the depth and breadth of this study, providing an initial rating, downgrading domains (risk of bias, inconsistency, indirectness, imprecision, and publication bias), upgrading domains (large effect or dose response, if confounders would reduce the effect), and an end certainty rating. Assessment was conducted independently by 2 reviewers. Heterogeneity and sensitivity analysis were not assessed within this review.

## Results

### Systematic Review Selection

**Figure 1.**
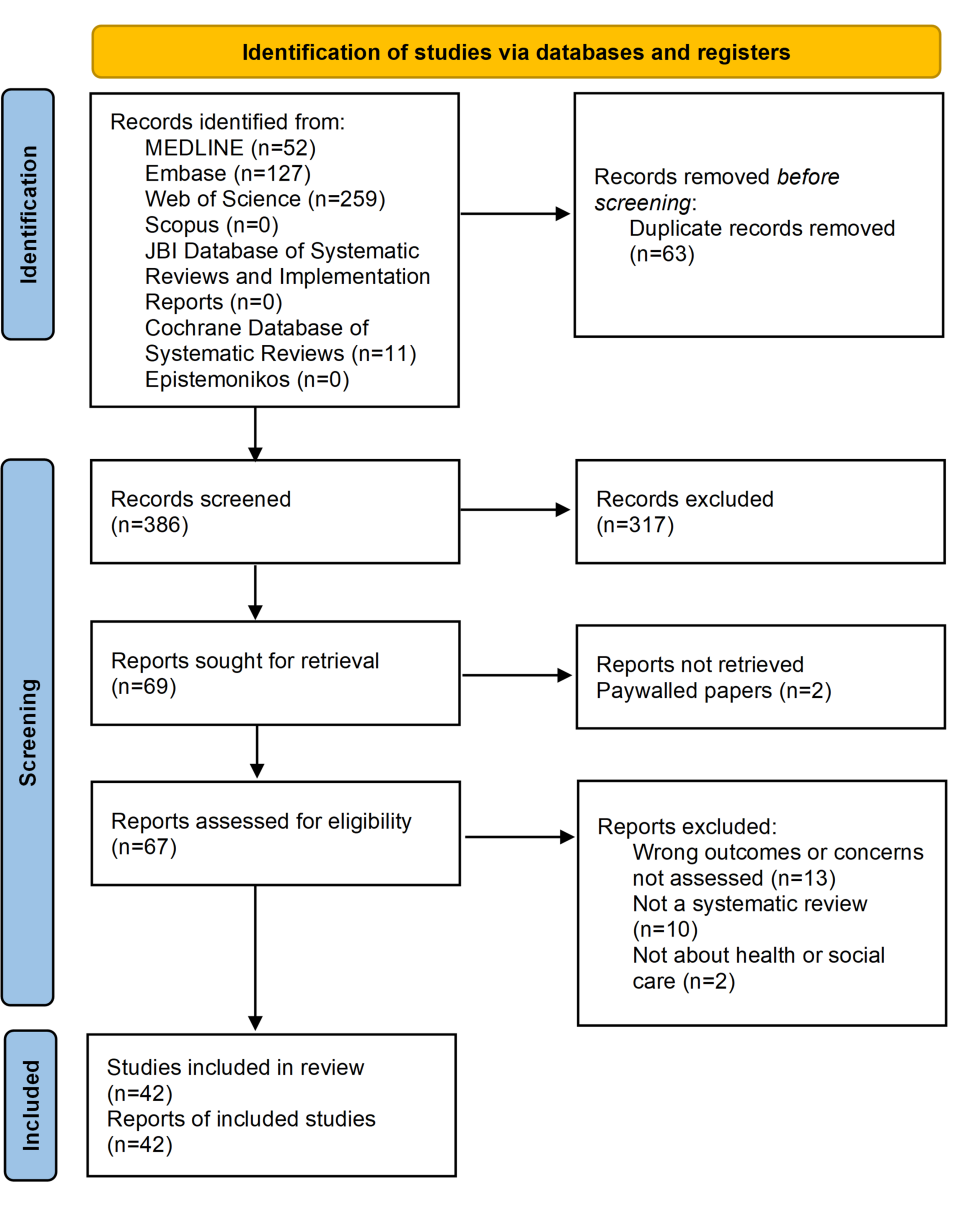
PRISMA flow diagram detailing numbers for study retrieval, screening, exclusion, and inclusion. PRISMA: Preferred Reporting Items for Systematic Reviews and Meta-Analyses.

In total, 449 records were identified from databases, as shown in the PRISMA flowchart (Figure 1).

Before screening, 63 duplicates were removed. The remaining 386 systematic reviews were initially screened by title and abstract. A total of 317 reviews that did not meet the inclusion criteria were excluded. The remaining 69 systematic reviews underwent an additional full-text screening. Further, 27 reports were excluded, most commonly due to concerns not being addressed, followed by topics being unrelated to health or social care, and not being a systematic review or being inaccessible behind a paywall (Figure 1 and Multimedia Appendix 2). A final total of 42 remaining systematic reviews were included in this umbrella review.

### Characteristics of Systematic Reviews

All studies identified were written in the English language. However, the country of the first author varied across the globe. Most systematic reviews originated from the United States (n=9) [10,36-43], followed by the United Kingdom (n=6) [44-49], Pakistan (n=4) [50-53], Australia (n=3) [12,54,55], Canada and Israel (n=2 each) [56-59], and 16 other countries with 1 systematic review each [9,11,60-73]. The most common year of publication was 2024, with 27 reviews, followed by 2023 with 9 reviews and 2025 with 6 reviews. No reviews were found before 2023 (Table 1).

The population raising concerns was as follows. Most commonly, it was researchers only (n=17) and other groups included clinicians (general; n=5), plastic surgeons (n=5), psychiatrists (n=4), and neurosurgeons (n=3). Less common were pediatricians, gastroenterologists, dermatologists, pathologists, cardiologists, ophthalmologists, orthopedics, and ICU nurses (n=1 each; Table 1).

The most common individual LLM was ChatGPT, with 15 systematic reviews focusing on this LLM alone [9,36,39,42,44,48,50,51,59-61,68,70-72]. Another 15 systematic reviews considered more than one LLM [10,12,37,38,40,45,46,49,54,56-58,62,65,67]. A smaller proportion of studies (n=12) examined LLMs within a broader context, such as AI in general [11,43,47,53,69,73], natural language processing [41,66], conversational agents [55,63], deep learning [52], and GenAI [64] (Table 1).

There was a positively skewed distribution of primary studies included in the systematic reviews. The IQR was between 19 and 83 (median 32; range 5‐315) studies. Further, 3 systematic reviews were classed as outliers as they examined higher numbers of primary studies, and a further 3 reviews did not share their sample size [39,58,65]. All systematic reviews were qualitative and did not perform a meta-analysis. Only 5.1% (n=2) formally tested for agreement between reviewers using Cohen κ (Multimedia Appendix 3 for full data extraction).

**Table 1.** Characteristics of the 39 included studies in this umbrella review, including AMSTAR-2^a^ and GRADE^b^ ratings for each included study.

Reviews	Country	AI^c^ of interest	Population raising concerns	Included studies (n)	AMSTAR-2 rating	GRADE rating
Abi-Rafeh et al [44], 2024	United Kingdom	ChatGPT (OpenAI)	Plastic surgeons	175	Critically low	Very low
Arif et al [50], 2024	Pakistan	ChatGPT	Plastic surgeons	32	Critically low	Very low
Balla et al [45], 2023	United Kingdom	AI in general - lists ChatGPT, Bard (Google LLC), and GLASS A.I. 2.0 (Glass Health)	Pediatricians	20	Critically low	Very low
Banskota et al [73], 2025	Nepal	AI (general)	Orthopedics	20	Critically low	Very low
Bečulić et al [60], 2024	Bosnia	ChatGPT	Neurosurgeons	13	Critically low	Very low
Fareed et al [53], 2025	Pakistan	LLMs^d^	Clinicians	27	Critically low	Very low
Fatima et al [51], 2024	Pakistan	ChatGPT	Clinicians	83	Critically low	Low
Garg et al [61], 2023	India	ChatGPT	Clinicians	118	Critically low	Very low
Guo et al [46], 2024	UK	LLMs: ChatGPT, BERT^e^	Researchers	40	Critically low	Low
Haltaufderheide and Ranisch [62], 2024	Germany	LLMs, ChatGPT	Researchers	53	Critically low	Low
Kiuchi et al [63], 2024	Japan	CAs^f^	Researchers	315	Critically low	Very low
Kiwan et al [47], 2024	United Kingdom	AI in general	Plastic surgeons	96	Critically low	Very low
Klang et al [36], 2023	United States	ChatGPT 3.5 (OpenAI)	Gastroenterologists	6	Critically low	Low
Kolding et al [64], 2024	Denmark	GenAI - includes ChatGPT	Psychiatrists	40	Critically low	Very low
Kucukkaya et al [9], 2024	Turkey	ChatGPT	ICU^g^ nurses	5	Critically low	Very low
Kutbi [67], 2024	Saudi Arabia	AI (general), LLMs	Researchers	19	Critically low	Very low
Li and Guenier [48], 2024	United Kingdom	ChatGPT 3.5 (4) (OpenAI)	Researchers	14	Critically low	Very low
Malgaroli et al [41], 2023	United States	NLP^h^	Psychiatrists	102	Critically low	Low
Mohamed et al [65], 2024	Oman	LLMs	Researchers	N/R^i^	Critically low	Low
Moya-Salazar et al [71], 2024	Peru	ChatGPT	Researchers	14	Critically low	Very low
Nasra et al [54], 2025	Australia	AI (general), LLMs	Clinicians	22	Critically low	Very low
Omar et al [56], 2024	Israel	LLMs	Psychiatrists	16	Low	Low
Omar et al [57], 2025	Israel	LLMs	Psychiatrists	34	Low	Low
Paganelli et al [66], 2024	Italy	NLP	Dermatologists	30	Critically low	Very low
Pashangpour and Nejat [58], 2024	Canada	LLMs	Researchers	N/R	Critically low	Very low
Patil et al [37], 2024	United States	LLMs	Neurosurgeons	51	Critically low	Very low
Pressman et al [10], 2024	United States	LLMs	Plastic surgeons	53	Critically low	Very low
Pressman et al [38], 2024	United States	LLMs	Plastic surgeons	34	Critically low	Very low
Rehman et al [52], 2025	Pakistan	Deep learning (including LLMs)	Researchers	100	Critically low	Very low
Roman et al [68], 2023	United Arab Emirates	ChatGPT	Neurosurgeons	22	Critically low	Very low
Rudnicka et al [69], 2024	Poland	AI (general)	Researchers	253	Critically low	Very low
Ruksakulpiwat et al [70], 2023	Thailand	ChatGPT	Researchers	6	Critically low	Very low
Sacoransky et al [59], 2024	Canada	ChatGPT	Researchers	8	Critically low	Very low
Sallam [72], 2023	Jordan	ChatGPT	Pathologists	60	Critically low	Very low
Sanjeewa et al [55], 2024	Australia	CAs	Researchers	19	Critically low	Low
Sharma et al [42], 2024	United States	ChatGPT	Cardiologists	24	Critically low	Very low
Tangsrivimol et al [39], 2025	United States	ChatGPT	Clinicians	N/R	Critically low	Very low
Villanueva-Miranda et al [43], 2025	United States	Deep learning	Researchers	83	Critically low	Very low
Wang et al [40], 2024	United States	LLMs - ChatGPT	Researchers	65	Critically low	Very low
Wangsa et al [12], 2024	Australia	ChatGPT, Bard, Llama (Meta AI), Ernie (Baidu), and Grok (xAI)	Researchers	28	Critically low	Very low
Wong et al [49], 2024	United Kingdom	LLMs	Ophthalmologists	32	Critically low	Very low
Younis et al [11], 2024	Iraq	AI (general)	Researchers	82	Critically low	Very low

a AMSTAR-2: A Measurement Tool to Assess Systematic Reviews.

b GRADE: Grading of Recommendations Assessment, Development, and Evaluation.

c AI: artificial intelligence.

d LLM: large language model.

e BERT: Bidirectional Encoder Representations from Transformers.

f CA: conversational agent.

g ICU: intensive care unit.

h NLP: natural language processing.

i N/R: not reported.

### Primary Study Overlap

Due to poor reporting of included primary studies within the systematic reviews, we were unable to calculate the corrected covered area to identify any overlap between primary studies. For example, some reviews listed the number of included studies but only referenced a subset in among supporting references, which meant the primary studies could not be easily identified. However, from manual inspection, it appears that primary studies that have been clearly reported were only included in a single systematic review. In either case, findings should be interpreted with caution as overinflation may be present.

### Quality Assessments

Regarding risk of bias in systematic reviews, the overall rating of reviews using AMSTAR-2 was either low (n=2, 4.8% reviews) [56,57] or critically low (n=40, 95.2% reviews) [9-11,36-55,58-73] (Table 1). A total of 40 (95.2%) reviews had a valid research question (Q1) [9-12,36-41,43-65,67-73], 24 (57.1%) reviews provided descriptions of their included studies (Q3) [9,11,36,38,40-42,44,46,50,51,54-57,60-62,64-66,68,70,72], and 27 (64.2%) reviews reported no conflicts of interest (Q16) [9-12,36-44,46-60,62,63,67-73].

However, only 6 (14.2%) reviews had a protocol (Q2) [41,46,56,57,62,68], 3 (7.1%) reviews justified their choice of included studies (Q8) [41,56,65], 10 (23.8%) reviews had a literature search strategy (Q4) [41,48,55-57,61,62,64,66,68], 1 (38%) review was completely double-screened (Q5) [10,36,40,41,46,48,49,55-57,61,64,68,70,71,73], 8 (19%) reviews were completely double data-extracted (Q6) [36,53,54,56,57,61,73], 1 (2.3%) review justified their exclusions (Q7) [46], 6 (14.2%) reviews had risk of bias assessment (Q9) [36,46,55-57,62], 6 (14.2%) reviews looked for funding disclosures of constituent studies (Q10) [9,36,43,49,53,73], 6 (14.2%) reviews accounted for risk of bias when interpreting results (Q13) [36,41,55-57,65], and 5 (11.9%) reviews addressed heterogeneity (Q14) [36,41,55,57,67]. No reviews completed a meta-analysis (Q11), a risk of bias assessment for a meta-analysis (Q12), or formally assessed for publication bias (Q15; Table 2).

**Table 2.** AMSTAR-2^a^ rating for each of the 39 included systematic reviews.

Reviews	1	2	3	4	5	6	7	8	9	10	11	12	13	14	15	16	Overall rating
Abi-Rafeh et al [44], 2024	Y^b^	N^c^	N	N	N	N	N	P^d^	N	N	N/A^e^	N/A	N	N	N	Y	Critically low
Arif et al [50], 2024	Y	N	N	N	N	N	N	P	N	N	N/A	N/A	N	N	N	Y	Critically low
Balla et al [45], 2023	Y	N	N	N	N	N	N	N	N	N	N/A	N/A	N	N	N	N	Critically low
Banskota et al [73], 2025	Y	P	N	N	Y	Y	N	P	P	Y	N/A	N/A	N	N	N	Y	Critically low
Bečulić et al [60], 2024	Y	N	N	N	N	N	N	P	N	N	N/A	N/A	N	N	N	Y	Critically low
Fareed et al [53], 2025	Y	N	N	P	N	Y	N	N	N	Y	N/A	N/A	N	N	N	Y	Critically low
Fatima et al [51], 2024	Y	N	N	N	N	N	N	P	N	N	N/A	N/A	N	N	N	Y	Critically low
Garg et al [61], 2023	Y	N	N	P	Y	Y	N	P	N	N	N/A	N/A	N	N	N	N	Critically low
Guo et al [46], 2024	Y	P	N	N	Y	N	N	P	Y	N	N/A	N/A	N	N	N	Y	Critically low
Haltaufderheide and Ranisch [62], 2024	Y	P	N	P	N	N	N	P	P	N	N/A	N/A	N	N	N	Y	Critically low
Kiuchi et al [63], 2024	Y	N	N	N	N	N	N	N	N	N	N/A	N/A	N	N	N	Y	Critically low
Kiwan et al [47], 2024	Y	N	N	N	N	N	N	N	N	N	N/A	N/A	N	N	N	Y	Critically low
Klang et al [36], 2023	Y	N	N	N	Y	Y	N	P	Y	Y	N/A	N/A	Y	Y	N	Y	Critically low
Kolding et al [64], 2024	Y	N	N	P	Y	N	N	P	N	N	N/A	N/A	N	N	N	N	Critically low
Kucukkaya et al [9], 2024	Y	N	N	N	N	N	N	P	N	Y	N/A	N/A	N	N	N	Y	Critically low
Kutbi [67], 2024	Y	N	N	N	N	N	N	N	N	N	N/A	N/A	N	Y	N	Y	Critically low
Li and Guenier [48], 2024	Y	N	N	P	Y	N	N	N	N	N	N/A	N/A	N	N	N	Y	Critically low
Malgaroli et al [41], 2023	Y	P	Y	P	Y	N	N	Y	N	N	N/A	N/A	Y	Y	N	Y	Critically low
Mohamed et al [65], 2024	Y	N	Y	N	N	N	N	P	N	N	N/A	N/A	Y	N	N	N	Critically low
Moya-Salazar et al [71], 2024	Y	N	N	N	Y	N	N	N	N	N	N/A	N/A	N	N	N	Y	Critically low
Nasra et al [54], 2025	Y	N	N	N	N	N	N	P	N	N	N/A	N/A	N	N	N	Y	Critically low
Omar et al [56], 2024	Y	P	Y	P	Y	Y	N	P	Y	N	N/A	N/A	Y	Y	N	Y	Low
Omar et al [57], 2025	Y	P	N	P	Y	Y	N	P	P	N	N/A	N/A	Y	N	N	Y	Low
Paganelli et al [66], 2024	Y	N	N	P	N	Y	N	P	N	N	N/A	N/A	N	N	N	N	Critically low
Pashangpour and Nejat [58], 2024	N	N	N	N	N	N	N	N	N	N	N/A	N/A	N	N	N	Y	Critically low
Patil et al [37], 2024	Y	N	N	N	N	N	N	N	N	N	N/A	N/A	N	N	N	Y	Critically low
Pressman et al [10], 2024	Y	N	N	N	Y	N	N	N	N	N	N/A	N/A	N	N	N	Y	Critically low
Pressman et al [38], 2024	Y	N	N	N	N	N	N	P	N	N	N/A	N/A	N	N	N	Y	Critically low
Rehman et al [52], 2025	Y	N	N	N	N	N	N	N	N	N	N/A	N/A	N	N	N	Y	Critically low
Roman et al [68], 2023	Y	P	N	P	Y	N	N	P	N	Y	N/A	N/A	N	N	N	Y	Critically low
Rudnicka et al [69], 2024	Y	N	N	N	N	N	N	N	N	N	N/A	N/A	N	N	N	Y	Critically low
Ruksakulpiwat et al [70], 2023	Y	N	N	N	Y	N	N	P	N	N	N/A	N/A	N	N	N	Y	Critically low
Sacoransky et al [59], 2024	Y	N	N	N	N	N	N	N	N	N	N/A	N/A	N	N	N	Y	Critically low
Sallam [72], 2023	Y	N	N	N	N	N	N	P	N	N	N/A	N/A	N	N	N	Y	Critically low
Sanjeewa et al [55], 2024	Y	N	N	P	Y	N	N	P	Y	N	N/A	N/A	Y	Y	N	Y	Critically low
Sharma et al [42], 2024	Y	N	N	N	N	N	N	P	N	N	N/A	N/A	N	N	N	Y	Critically low
Tangsrivimol et al [39], 2025	N	N	N	N	N	N	N	N	N	N	N/A	N/A	N	N	N	Y	Critically low
Villanueva-Miranda et al [43], 2025	Y	N	N	P	N	N	N	P	N	Y	N/A	N/A	N	N	N	Y	Critically low
Wang et al [40], 2024	Y	N	N	N	Y	N	N	P	N	N	N/A	N/A	N	N	N	Y	Critically low
Wangsa et al [12], 2024	Y	N	N	N	N	N	N	N	N	N	N/A	N/A	N	N	N	Y	Critically low
Wong et al [49], 2024	Y	N	N	N	Y	N	N	N	N	N	N/A	N/A	N	N	N	Y	Critically low
Younis et al [11], 2024	Y	N	N	N	N	N	N	P	N	N	N/A	N/A	N	N	N	Y	Critically low

a AMSTAR-2: A Measurement Tool to Assess Systematic Reviews.

b Y: yes.

c N: no.

d P: partial.

e N/A: not applicable.

### Certainty of Evidence

Reviews were overall graded as either low-end certainty (n=9, 21.4%) [36,41,46,51,55-57,62,65], or very low-end certainty (n=33, 78.6%) [9-12,37-40,42-44,47-50,52-54,58-61,63,66-73]. As there were no randomized controlled trials, all reviews started as low certainty before downgrading or upgrading (initial rating).

In the downgrading domains, all studies had consistent results (inconsistency) [9-12,36-73] and most addressed the core question (indirectness) of this umbrella review (n=36, 85.7%) [9-12,36-40,42,44-46,48-51,53-62,64-68,70-73]. However, only 7 (16.6%) reviews assessed for risk of bias [36,46,52,55-57,73], 14 (33.3%) reviews directly addressed the research question (imprecision) [10,12,36,40,43,46,48,51,53,56,57,62,65,73], and 4 (9.5%) reviews considered publication bias [41,51,62,65].

In terms of upgrading domains, 8 (19%) reviews were considered as showing large effects [10,36,46,51,56,57,62,65], but only 1 (2.3%) review mentioned confounders [41]. None displayed a dose-response relationship, and as this criterion was not relevant to this review, it was not taken into account for the final scoring (Table 3).

**Table 3.** GRADE^a^ assessment for each of the 39 included systematic reviews.

				Downgrading domains					Upgrading domains			
Reviews	Depth of concerns	Breadth of concerns	Initial rating	RoB^b^	Inconsistency	Indirectness	Imprecision	Publication bias	Large effect	Dose response	Confounders would reduce the effect	End certainty rating
Abi-Rafeh et al [44], 2024	Y^c^	Y	Low	N^d^	Y	Y	N	N	N	N	N	Very low
Arif et al [50], 2024	N	Y	Low	N	Y	Y	N	N	N	N	N	Very low
Balla et al [45], 2023	Y	N	Low	N	Y	Y	N	N	N	N	N	Very low
Banskota et al [73], 2025	Y	Y	Low	Y	Y	Y	Y	N	N	N	N	Very low
Bečulić et al [60], 2024	N	N	Low	N	Y	Y	N	N	N	N	N	Very low
Fareed et al [53], 2025	Y	N	Low	N	Y	Y	Y	N	N	N	N	Very low
Fatima et al [51], 2024	Y	Y	Low	N	Y	Y	Y	Y	Y	N	N	Low
Garg et al [61], 2023	N	Y	Low	N	Y	Y	N	N	N	N	N	Very low
Guo et al [46], 2024	Y	Y	Low	Y	Y	Y	Y	N	Y	N	N	Low
Haltaufderheide and Ranisch [62], 2024	Y	Y	Low	N	Y	Y	Y	Y	Y	N	N	Low
Kiuchi et al [63], 2024	Y	Y	Low	N	Y	N	N	N	N	N	N	Very low
Kiwan et al [47], 2024	N	N	Low	N	Y	N	N	N	N	N	N	Very low
Klang et al [36], 2023	Y	N	Low	Y	Y	Y	Y	N	Y	N	N	Low
Kolding et al [64], 2024	N	Y	Low	N	Y	Y	N	N	N	N	N	Very low
Kucukkaya et al [9], 2024	Y	Y	Low	N	Y	Y	N	N	N	N	N	Very low
Kutbi [67], 2024	Y	Y	Low	N	Y	Y	N	N	N	N	N	Very low
Li and Guenier [48], 2024	Y	Y	Low	N	Y	Y	Y	N	N	N	N	Very low
Malgaroli et al [41], 2023	Y	Y	Low	N	Y	N	N	Y	Y	N	Y	Low
Mohamed et al [65], 2024	Y	Y	Low	N	Y	Y	Y	Y	Y	N	N	Low
Moya-Salazar et al [71], 2024	N	N	Low	N	Y	Y	N	N	N	N	N	Very low
Nasra et al [54], 2025	N	N	Low	N	Y	Y	N	N	N	N	N	Very low
Omar et al [56], 2024	Y	N	Low	Y	Y	Y	Y	N	Y	N	N	Low
Omar et al [57], 2025	N	N	Low	Y	Y	Y	Y	N	Y	N	N	Low
Paganelli et al [66], 2024	N	N	Low	N	Y	Y	N	N	N	N	N	Very low
Pashangpour and Nejat [58], 2024	Y	Y	Low	N	Y	Y	N	N	N	N	N	Very low
Patil et al [37], 2024	Y	Y	Low	N	Y	Y	N	N	N	N	N	Very low
Pressman et al [10], 2024	Y	Y	Low	N	Y	Y	Y	N	Y	N	N	Very low
Pressman et al [38], 2024	N	Y	Low	N	Y	Y	N	N	N	N	N	Very low
Rehman et al [52], 2025	N	N	Low	Y	Y	N	N	N	N	N	N	Very low
Roman et al [68], 2023	N	N	Low	N	Y	Y	N	N	N	N	N	Very low
Rudnicka et al [69], 2024	Y	N	Low	N	Y	N	N	N	N	N	N	Very low
Ruksakulpiwat et al [70], 2023	N	Y	Low	N	Y	Y	N	N	N	N	N	Very low
Sacoransky et al [59], 2024	N	N	Low	N	Y	Y	N	N	N	N	N	Very low
Sallam [72], 2023	Y	Y	Low	N	Y	Y	N	N	N	N	N	Very low
Sanjeewa et al [55], 2024	N	N	Low	Y	Y	Y	N	N	N	N	N	Low
Sharma et al [42], 2024	N	N	Low	N	Y	Y	N	N	N	N	N	Very low
Tangsrivimol et al [39], 2025	Y	Y	Low	N	Y	Y	N	N	N	N	N	Very low
Villanueva-Miranda et al [43], 2025	Y	N	Low	N	Y	N	Y	N	N	N	N	Very low
Wang et al [40], 2024	Y	Y	Low	N	Y	Y	Y	N	N	N	N	Very low
Wangsa et al [12], 2024	Y	Y	Low	N	Y	Y	Y	N	N	N	N	Very low
Wong et al [49], 2024	N	Y	Low	N	Y	Y	N	N	N	N	N	Very low
Younis et al [11], 2024	Y	Y	Low	N	Y	Y	N	N	N	N	N	Very low

a GRADE: Grading of Recommendations Assessment, Development, and Evaluation.

b RoB: Risk of Bias.

c Y: Yes.

d N: No.

### Interrater Reliability

κ statistics were calculated between the reviewers (FY and one of PA, MF, or HO). Significant agreement was seen for both AMSTAR-2 (0.92, indicating near-perfect agreement) and GRADE (0.75, indicating substantial agreement). The κ statistic was considered significant where it was 0.6 (substantial agreement) or higher (Table 4) [74].

**Table 4.** Interrater reliability, Cohen κ, detailing the agreement rates between reviewers when assessing risk of bias (AMSTAR-2^a^) and certainty of the evidence (GRADE^b^).

Reviewer combination	AMSTAR-2	GRADE
FY and PA	0.88^c^	0.75^d^
FY and MF	0.88^c^	0.5
FY and HO	1.00^c^	1.00^c^
Average	0.92^c^ (SD 0.05)	0.75^d^ (SD 0.2)

a AMSTAR-2: A Measurement Tool to Assess Systematic Reviews.

b GRADE: Grading of Recommendations Assessment, Development, and Evaluation.

c Shows statistical significance of near-perfect agreement between reviewers.

d Shows statistical significance of substantial agreement.

### Synthesis of Results

Qualitative coding is a means of deriving descriptive tags to categorize data, which can then be used to generate themes. A total of 29 codes were generated from the synthesis (Multimedia Appendix 3), and seven themes emerged: (1) data quality and reliability; (2) transparency and reproducibility; (3) performance and capability; (4) technical and operational; (5) human interaction and social impact; (6) ethical, legal, and safety; and (7) costs. These could be grouped under 3 core themes: technical capability; ethical, legal, and societal; and costs. For most population groups, mentions of technical capability concerns were the greatest, followed by ethical, legal, and societal concerns, and then cost concerns (Table 5).

**Table 5.** Count of systematic reviews raising concerns by population groups under the themes derived from qualitative thematic analysis.

	Cardio^a^ (1)	Derma^b^ (1)	Gastro^c^ (1)	GC^d^ (5)	ICU^e^ nurses (1)	Neuro^f^ (3)	Ophtha^g^ (1)	Ortho^h^ (1)	Pedia^i^ (1)	Patho^j^ (1)	Psurg^k^ (5)	Psychia^l^ (4)	Resea^m^ (17)
Technical capabilities													
Data quality and reliability	1	1	1	5	0	3	1	1	1	1	5	4	15
Transparency and reproducibility	1	0	0	3	0	3	0	1	1	1	3	2	8
Performance and capability	1	1	1	5	0	2	1	1	1	1	4	4	12
Technical and operations	0	0	0	1	0	1	0	1	0	0	0	1	6
Ethical, legal, and societal													
Human interaction and social impact	0	1	0	5	1	1	0	0	0	1	5	2	12
Legal, ethical, and safety	1	1	1	5	1	3	1	1	1	1	5	4	16
Costs													
All costs	0	0	0	1	0	1	0	1	0	0	0	0	4

a Cardio: cardiologists.

b Derma: dermatologists.

c Gastro: gastroenterologists.

d GC: general clinicians.

e ICU: intensive care unit.

f Neuro: neurosurgeons.

g Ophtha: ophthalmologists.

h Ortho: orthopedics.

i Pedia: pediatricians.

j Patho: pathologists.

k Psurg: plastic surgeons.

l Psychia: psychiatrists.

m Resea: researchers.

### Data Quality and Reliability

The 42 studies reflected that bias in training algorithms and datasets mirrors what is already known on the subject, with estimates that around a quarter of studies on LLMs show bias [40]. For sensitive topics, this may be reduced to around 15% [65]. Key demographic barriers were mentioned, including sex, race, culture, language, and religion [43,55,61]. Political biases were also highlighted in closed-source algorithms [12]. Specifically, racial biases affecting individuals of black ethnicity were mentioned [62]. Sexual discrimination, bias toward female doctors was highlighted, with AI recommending fewer female doctors than male doctors. In pediatric medicine, a key issue was a limited, fragmented, or total lack of standardized training sets for LLMs in genetic disorders [45]. Most genetic disorders are rare and disproportionately affect children, which may contribute to the incomplete training sets [75].

Furthermore, outdated and limited datasets were commonly mentioned, with the date cutoffs highlighted for various ChatGPT models. For example, ChatGPT 3.5 was pretrained until September 2021 only and did not incorporate information from the internet [11,76]. ChatGPT 4 was pretrained up until April 2023 only [46,59,73]. Obvious problems with this include a lack of recent knowledge, obsolete knowledge, or misalignment with current clinical guidelines [50,53]. Another study also acknowledged that hospital-specific protocols should be adhered to, which LLMs may not include in their outputs [9]. From clinical practice, trust guidelines may differ from general ones, particularly in the case of antimicrobials, as location affects the presence of different microbes [77].

Fabricated or fake references were highlighted in 4 of the reviews [40,50,51,54]. Constituent studies that examined image references were anatomically incorrect or fabricated in 81% of cases [9]. Model overfitting, where a model is trained too specifically so that it cannot generalize on unseen data, was explored in reviews that examine image-based medical applications. Radiologically, x-ray images may be prone to overfitting [52]. Dermatology, which relies heavily on pattern recognition, may also be prone to this phenomenon [66]. Disease management options often featured fabricated references in all versions of ChatGPT [59]. Furthermore, the potential for such references to mislead junior colleagues, including resident doctors, was mentioned [59]. Similarly, several studies mentioned that hallucinations were a cause of concern [39,43,51,53,58,64]. Some studies emphasized that it was easy to be convinced by hallucinations [51,64]. Other aspects addressed included that hallucinations were often confidently given by LLMs, especially when parameter settings that encourage more varied, random outputs are used [46]. Other factors that influenced hallucination generation were the quality of prompts, with prompts phrased as stories often causing hallucinations [58]. It was estimated in 1 review that 40% of discharge summaries written by AI have hallucinations [39].

### Transparency and Reproducibility

Several studies highlight the difficulties with the transparency of LLMs, with some referring to the technology as a “black box” [37,43,51,56,69,73]. Ethical discussions are highlighted as a way to build transparency of models [10]. Profit-driven lack of transparency was only mentioned in 1 review, but another highlighted how the proprietary nature of LLMs can complicate openness and trust [53,62]. Another review emphasized that a lack of transparency can be ascribed to multiple stages, including the design and training of LLMs [65]. The same review also emphasized that care should be taken so that interventions aimed at increasing transparency do not inadvertently expose patient data. Leaked personal data can potentially also amplify bias through normalizing biased correlations as real patterns [65].

The issue of repeatability with the same or similar prompts was also isolated as a concern. Most constituent primary research papers were identified as prompt experiments [64]. However, Patil et al [37] reported that about a quarter of the research papers were found not to disclose their prompts. Another review underlined that when they were disclosed, prompts tended to be single, standalone 1-shot prompts [59]. The concept of biased prompts was raised, and prompt injection, where prompts are engineered to extract information or cause disruption for nefarious purposes, was also highlighted as a concern [36,43].

### Performance and Capability

The reviews in this study broadly agreed that the performance of LLMs was comparable to or exceeded that of humans. Latest estimates of correct responses on neurology licensing examination questions revealed ChatGPT 4 had 85% accuracy compared to human performance at 73.8% [46]. Fracture detection rates with deep learning were also close to this figure at 83% [67]. Approximately 76.5% (32/42) of studies used human performance as a benchmark, and 50% (21/42) compared LLMs to human performance alone. It is essential to measure success on diverse tasks, and some reviews agreed that LLMs often struggle with complexity [11,39,45,69,72]. Lack of originality was also a commonly cited concern [11,36,44,51,61,70,72]. Most systematic reviews emphasized that LLMs needed human oversight, and several mentioned it should only be viewed as a “supplementary tool” [9,39,45,50,56,57,60].

### Technical and Operational Challenges

Few studies explored technical and operational challenges. Further, 1 review cited a primary study that examined system crashes [48]. Another review highlighted slow response times and how this was a problem in robotics [58]. Similarly, a second review noted slower clinical workflows due to the verification process [53]. Aside from the speed of development outstripping research, it was noted that a lack of research was problematic [64]. Another review quoted that only 26% of studies used randomized controlled trial–type designs in assessing LLMs by users, suggesting that conclusions from research may not be completely reliable [55]. Additionally, 1 review called for rigorous validation in real-world settings, which was supported by another review raising gaps in validation as a concern [43,73].

### Human Interaction and Social Impact

There was a mix of views regarding empathy and LLMs. Some reviews stressed that LLMs’ empathy was good, although it was usually assessed through technical expert reports rather than by both users and experts. Only a minority of studies (n=10) used an explicit definition of the term “empathy” [46,54,55]. Others stated there was a general lack of empathy [11,39,50,64]. Only 1 study gave a mix of such opinions [47].

Furthermore, some reviews expressed that LLMs could cause potential damage to the doctor-patient relationship [38,44,46,62]. There was a large overlap between this finding and reviews that addressed deskilling of the workforce or impacts on the job market [11,44,46,62,65]. Additionally, a lack of a sustainable relationship between new technologies and the user was emphasized in robotics due to problems with “semantics, consistency, and interactiveness” [63]. Researchers raised concerns around challenges with linguistic complexity, such as understanding irony and sarcasm [43].

User acceptance was identified as an important factor in 2 reviews [46,67]. The importance of acceptance by various groups, including physicians, caregivers, and providers, was highlighted [63]. Potential mistrust of LLMs and humanization issues were highlighted in the mental health field [43,55]. Yet, public acceptance was viewed as very positive in 1 systematic review [40]. There was an acknowledgment that this area needs more research [48].

Further, 5 reviews emphasized that existing inequalities could become more entrenched with LLMs [40,47,62,65,70]. This is separate from concerns over bias, which could also contribute to the deepening of inequalities. Both of these events could perpetuate the other, whereby biased outputs could deepen inequalities, which in turn could lead to the introduction of further biases.

### Legal, Ethical, and Safety Concerns

Almost all reviews mentioned ethical concerns as a potential problem with LLM use, with varying degrees of explanation on this topic. Accountability was addressed from a 2-fold perspective, with 4 reviews focusing on medicolegal accountability [11,44,59,72]. Others were focused on legitimacy and accountability in research [50,51]. Suggested solutions included clear guidelines on accountability [12,48,55]. Another potential solution was policy and regulations [40,65]. Related to this was the issue of academic integrity, alternatively phrased as “pedagogical risk” [9,11,12,44,62,65,68].

Most reviews raised privacy and security concerns regarding LLMs. The privacy problems highlighted by studies can be seen as a triad involving information leaks from embedded training examples, inferential disclosure, and insufficiently deidentified data [40]. There may be a trade-off between data utility and privacy [40,65]. The need to comply with existing laws, including GDPR, was emphasized by 3 systematic reviews [9,12,59]. Other studies acknowledged that further efforts were necessary, such as limiting personal data collection or conducting audits [39,40,55,73].

Only 9 reviews discussed obtaining consent to collect personal data [10,12,37,39,43,44,53,62,73]. This reflects the limited literature available on ensuring informed consent when using LLMs and highlights the fact that new technology is outpacing safety. Just 1 review sought to explain consent itself, which entails providing a full disclosure of the risks and benefits in such a way that the participant comprehends and agrees [10]. Further, 2 reviews suggested that protocols are needed to obtain consent [12,39]. Consent is crucial in anticipation of seen and unforeseen ethical issues with LLMs, and yet other systematic reviews chose to discuss LLMs as a way to streamline consent forms [37,44,62].

Safety may become a population-wide problem as well as an individual one. Mass-scale problems may become apparent with the potential of infodemics perpetuated by LLMs, according to some reviews [51,53,62,65,72]. The saturation of scientific literature with low-quality automated reviews, which may fuel infodemics, was also discussed [62]. In a pandemic context such as COVID-19, there could be even greater consequences [65].

Finally, the risk of self-propagating and uncontrolled evolution was described as “unknown” by 1 review in passing [44]. Self-propagating and uncontrolled evolution relates to the LLM’s ability to grow and change on its own. Considering that this could be very detrimental and have long-term impacts, this is important. While this possibility has been informally mentioned in technology circles for some time, there are only a small number of new reports in the literature examining this possibility, so this finding is expected.

### Environmental, Processing, and Economic Costs

None of the reviews examined more than one cost aspect, and most lacked depth of answers in relation to this topic. Costs were broadly environmental related to computational processing [43,58,65]. Given the scale of climate change and its importance to health, this is an area of great interest and should be explored further [78,79]. Other costs mentioned were financial costs incurred by users [40,60].

## Discussion

### Principal Findings

This umbrella review aimed to narratively synthesize the concerns that health care professionals and researchers face when using AI. A wide variety of concerns are raised, which overlap and interlink, consistently affecting multiple populations. The findings indicate 3 core areas of shared concern within the health care field: technical capability of AI; ethical, legal, and societal implications for use; and associated costs.

Much of the concern surrounding technical capability lies with data quality and reproducibility. For research to be as robust as possible, we must use AI for tasks that are appropriate, judicious, and sense-checked, as well as monitoring the functions and effects of LLMs [38,39]. A good starting point is to acknowledge where, how, and when AI was used. Research integrity policies, reporting guidelines, and audits will be crucial in meeting quality standards and enabling reproducibility [9,37,40,65,73]. Most of the reviews included here advocated for guidelines, although none explored direct examples or attempted to construct a guideline. This is expected, given that international and national guidelines are only gradually emerging. However, it could be argued that research should be a proactive driver for exploring these issues rather than a reactive one.

Ethical, legal, and societal implications were varied and broad-ranging. Concerns over hallucinations, bias, inequalities, and consent provide interesting and often deeply interrelated perspectives that triangulate with the technical capabilities of AI. For instance, bias that perpetuates “hallucinations” was commonly cited [61,62,72]. Efforts for transparency may inadvertently perpetuate bias, as implicit biases in models can exist even when they show no explicit biases [80]. This gives a false sense of security surrounding systems that are lacking in objectivity. The reuse of biased datasets from such models will worsen this problem, and further research is needed to explore these perpetuating cycles. Ultimately, transparency must be judged by humans who may reinforce biases inadvertently or fail to acknowledge problems because features are unreported or untested. Therefore, while we should strive for more transparency, particularly through legislation, guidance, and reporting, we should avoid labeling any LLM as completely “transparent.”

Some biases were thought to be pervasive, for instance, “social biases entrenched in data.” Specifically, female ophthalmologists were recommended less than one-third as often as their male counterparts [47]. Biases were present in topical issues; for example, “unfavorable attitudes” were described when ChatGPT was prompted to discuss topics such as climate change and Black Lives Matter [65]. Research is uncovering both implicit and overt biases toward already disadvantaged populations, which is why careful consideration of LLMs and their applications is necessary to prevent exacerbating existing health inequalities. Education surrounding considerations of equality, diversity, and inclusion when using LLMs may be helpful on an individual level. However, discriminatory biases may only be overcome by careful curation of training data underpinning the LLMs.

Some terms regarding LLMs could be revised for clarity and to destigmatize. Findings indicate the term “hallucination” for AI issues is considered unhelpful and stigmatizing for those with a psychiatric disorder, reflecting proposals in the literature for the term “AI misinformation” [64,81]. The term “hallucination” was originally used in computing science to refer to retained outputs even when artificial neural networks were pruned by removing some connections [82]. The term has evolved, first to positively describe tasks related to computer vision and improved facial recognition, and then to mean the generation of incorrect outputs in translation or object detection. Currently, it is used to mean incorrect LLM outputs produced with confidence [83]. As an overall term to describe confidently produced errors, AI “hallucination” may, on the surface, be useful. However, LLMs are not produced the same way as biological hallucination, which occurs in the absence of external stimuli [84]. By contrast, LLMs have external stimuli in the form of training data and prompts but still produce nonsensical outputs. This can be stigmatizing for those with a psychiatric disorder and is technically imprecise. AI generalization, fact fabrication, or stochastic parroting could be used as more distinct terms depending on the types of error seen [83].

Interestingly, while cost was a core theme, there was no definitive cost element that was unifying across reviews. Further explorations of cost concerns could inform cost-benefit analyses and full economic evaluations of AI use cases. We have seen such evaluations for AI-assisted health care technologies, but not regarding AI use in general health care practice and research [85].

### Limitations of This Review

The constituent systematic reviews comprised primary research papers that were heterogeneous. For example, different papers used different methods of evaluation in 1 review, ranging from surveys to response ratings and interview feedback [55]. Moreover, the authors used different methods to classify the accuracy of LLMs and did not adhere to standard formal procedures for assessment [40]. The quality of the included reviews was generally poor, and the extent of publication bias was unknown. Many of the poorly reported reviews included studies, meaning we were unable to determine the overlap between primary studies using the corrected covered area. Caution should be taken that different populations’ views of LLM limitations are not a complete representation of views of the broader population, and that findings may be overinflated due to the unknown overlap of primary studies.

Furthermore, thematic analyses have an element of subjectivity. Potential sources of bias include researcher bias and confirmation bias, whereby preexisting beliefs and experiences may have influenced the coding. We have attempted to limit this through group discussion of codes; however, future studies could incorporate a blinded dual coding process.

This review is also limited by the search dates of the included systematic reviews. As the field of LLMs rapidly evolves, primary studies published after the search dates may provide valuable insights into current thinking.

### Conclusions

To our knowledge, this is the first umbrella review to address the concerns of LLMs in health care research and practice. Thematic analyses provided insight into the complexity of different perspectives, and by using a whole population approach, it demonstrates common narratives. However, the poor quality of the included studies is a substantial limitation, and results should be interpreted with caution. Data quality is at the heart of these concerns, and combative action must ensure health care professionals and researchers have the resources required to overcome these apprehensions if AI is to be used routinely. Ethical, legal, and societal implications of AI use were also commonly raised. As technology accelerates and demands on health care increase, we must adapt and embrace change with equity, diversity, inclusion, and safety at the core.

## Supplementary material

10.2196/87804 Multimedia Appendix 1 Search strategies.

10.2196/87804 Multimedia Appendix 2 Excluded studies.

10.2196/87804 Multimedia Appendix 3 Data extraction and thematic coding.

10.2196/87804 Checklist 1 PRIOR checklist.

10.2196/87804 Checklist 2 PRISMA-S checklist.

10.2196/87804 Checklist 3 PRISMA 2020 abstract checklist.

## Abbreviations

AI: artificial intelligence
AMSTAR-2: A Measurement Tool to Assess Systematic Reviews
DOI: Digital Object Identifier
GDPR: General Data Protection Regulation
GenAI: generative artificial intelligence
GRADE: Grading of Recommendations Assessment, Development, and Evaluation
LLM: large language model
NICE: National Institute of Health and Care Excellence
PRIOR: Preferred Reporting Items for Systematic Reviews and Meta-Analyses
PRISMA: Preferred Reporting Items for Systematic Reviews and Meta-Analyses
PRISMA-S: Preferred Reporting Items for Systematic Reviews and Meta-Analyses - Search
SPIDER: Sample, Phenomenon of Interest, Design, Evaluation, Research Type

## References

[1] Batko K, Ślęzak A (2022). The use of big data analytics in healthcare. J Big Data.

[2] Rathore MM, Shah SA, Shukla D, Bentafat E, Bakiras S (2021). The role of ai, machine learning, and big data in digital twinning: a systematic literature review, challenges, and opportunities. IEEE Access.

[3] Goel A, Gueta A, Gilon O (2023). LLMs accelerate annotation for medical information extraction. arXiv.

[4] Ferraris AF, Audrito D, Caro LD, Poncibò C (2025). The architecture of language: understanding the mechanics behind LLMs. Camb Forum AI Law Gov.

[5] Hufton AL (2023). No artificial intelligence authors, for now. Patterns.

[6] (2024). Use of AI in evidence generation: NICE position statement. National institute of Health and Care Excellence.

[7] (2023). EU AI act: first regulation on artificial intelligence. European Parliament.

[8] The Artificial Intelligence and Data Act (AIDA) – companion document. Government of Canada.

[9] Kucukkaya A, Arikan E, Goktas P (2024). Unlocking ChatGPT’s potential and challenges in intensive care nursing education and practice: a systematic review with narrative synthesis. Nurs Outlook.

[10] Pressman SM, Borna S, Gomez-Cabello CA, Haider SA, Haider C, Forte AJ (2024). AI and ethics: a systematic review of the ethical considerations of large language model use in surgery research. Healthcare (Basel).

[11] Younis HA, Eisa TAE, Nasser M (2024). A systematic review and meta-analysis of artificial intelligence tools in medicine and healthcare: applications, considerations, limitations, motivation and challenges. Diagnostics (Basel).

[12] Wangsa K, Karim S, Gide E, Elkhodr M (2024). A systematic review and comprehensive analysis of pioneering AI chatbot models from education to healthcare: ChatGPT, Bard, Llama, Ernie and Grok. Future Internet.

[13] Meng X, Yan X, Zhang K (2024). The application of large language models in medicine: a scoping review. iScience.

[14] Li J, Zhou Z, Lyu H, Wang Z (2025). Large language models-powered clinical decision support: enhancing or replacing human expertise?. Intell Med.

[15] Azam M, Chen Y, Arowolo MO, Liu H, Popescu M, Xu D (2024). A comprehensive evaluation of large language models in mining gene interactions and pathway knowledge. bioRxiv.

[16] Ahn S (2024). The transformative impact of large language models on medical writing and publishing: current applications, challenges and future directions. Korean J Physiol Pharmacol.

[17] Lin C, Kuo CF (2025). Roles and potential of large language models in healthcare: a comprehensive review. Biomed J.

[18] Ganzinger M, Kunz N, Fuchs P (2025). Automated generation of discharge summaries: leveraging large language models with clinical data. Sci Rep.

[19] Qin H, Tong Y (2025). Opportunities and challenges for large language models in primary health care. J Prim Care Community Health.

[20] Jenkins K (2025). 10 year health plan for England. J Kidney Care.

[21] Cohen AM, Hersh WR, Peterson K, Yen PY (2006). Reducing workload in systematic review preparation using automated citation classification. J Am Med Inform Assoc.

[22] Wallace BC, Trikalinos TA, Lau J, Brodley C, Schmid CH (2010). Semi-automated screening of biomedical citations for systematic reviews. BMC Bioinformatics.

[23] Eastaugh CH, Still M, Beyer FR, Wallace SA, O’Keefe H (2025). Exploring the role of artificial intelligence in evidence synthesis: insights from the CORE Information Retrieval Forum 2025. Cochrane Evid Synth Methods.

[24] (2025). Safety and security risks of generative artificial intelligence to 2025 (Annex B). GOV.UK.

[25] Tredinnick L, Laybats C (2023). The dangers of generative artificial intelligence. Bus Inf Rev.

[26] von Eschenbach WJ (2021). Transparency and the black box problem: why we do not trust AI. Philos Technol.

[27] Dhar P (2020). The carbon impact of artificial intelligence. Nat Mach Intell.

[28] Page MJ, McKenzie JE, Bossuyt PM (2021). The PRISMA 2020 statement: an updated guideline for reporting systematic reviews. BMJ.

[29] Gates M, Gates A, Pieper D (2022). Reporting guideline for overviews of reviews of healthcare interventions: development of the PRIOR statement. BMJ.

[30] Rethlefsen ML, Kirtley S, Waffenschmidt S (2021). PRISMA-S: an extension to the PRISMA Statement for Reporting Literature Searches in Systematic Reviews. Syst Rev.

[31] Campbell M, McKenzie JE, Sowden A (2020). Synthesis without meta-analysis (SWiM) in systematic reviews: reporting guideline. BMJ.

[32] Ouzzani M, Hammady H, Fedorowicz Z, Elmagarmid A (2016). Rayyan-a web and mobile app for systematic reviews. Syst Rev.

[33] Prasad M (2024). Introduction to the GRADE tool for rating certainty in evidence and recommendations. Clin Epidemiol Glob Health.

[34] Shea BJ, Reeves BC, Wells G (2017). AMSTAR 2: a critical appraisal tool for systematic reviews that include randomised or non-randomised studies of healthcare interventions, or both. BMJ.

[35] Braun V, Clarke V (2006). Using thematic analysis in psychology. Qual Res Psychol.

[36] Klang E, Sourosh A, Nadkarni GN, Sharif K, Lahat A (2023). Evaluating the role of ChatGPT in gastroenterology: a comprehensive systematic review of applications, benefits, and limitations. Ther Adv Gastroenterol.

[37] Patil A, Serrato P, Chisvo N, Arnaout O, See PA, Huang KT (2024). Large language models in neurosurgery: a systematic review and meta-analysis. Acta Neurochir (Wien).

[38] Pressman SM, Borna S, Gomez-Cabello CA, Haider SA, Haider CR, Forte AJ (2024). Clinical and surgical applications of large language models: a systematic review. J Clin Med.

[39] Tangsrivimol JA, Darzidehkalani E, Virk HUH (2025). Benefits, limits, and risks of ChatGPT in medicine. Front Artif Intell.

[40] Wang L, Wan Z, Ni C (2024). Applications and concerns of ChatGPT and other conversational large language models in health care: systematic review. J Med Internet Res.

[41] Malgaroli M, Hull TD, Zech JM, Althoff T (2023). Natural language processing for mental health interventions: a systematic review and research framework. Transl Psychiatry.

[42] Sharma A, Medapalli T, Alexandrou M, Brilakis E, Prasad A (2024). Exploring the role of ChatGPT in cardiology: a systematic review of the current literature. Cureus.

[43] Villanueva-Miranda I, Xie Y, Xiao G (2025). Sentiment analysis in public health: a systematic review of the current state, challenges, and future directions. Front Public Health.

[44] Abi-Rafeh J, Xu HH, Kazan R, Tevlin R, Furnas H (2024). Large language models and artificial intelligence: a primer for plastic surgeons on the demonstrated and potential applications, promises, and limitations of ChatGPT. Aesthet Surg J.

[45] Balla Y, Tirunagari S, Windridge D (2023). Pediatrics in artificial intelligence era: a systematic review on challenges, opportunities, and explainability. Indian Pediatr.

[46] Guo Z, Lai A, Thygesen JH, Farrington J, Keen T, Li K (2024). Large language models for mental health applications: systematic review. JMIR Ment Health.

[47] Kiwan O, Al-Kalbani M, Rafie A, Hijazi Y (2024). Artificial intelligence in plastic surgery, where do we stand?. JPRAS Open.

[48] Li M, Guenier AW (2024). ChatGPT and health communication: a systematic literature review. IJEHMC.

[49] Wong M, Lim ZW, Pushpanathan K (2024). Review of emerging trends and projection of future developments in large language models research in ophthalmology. Br J Ophthalmol.

[50] Arif F, Safri MK, Shahzad Z, Yasmeen SF, Rahman MF, Shaikh SA (2024). Exploring the application of CHATGPT in plastic surgery: a comprehensive systematic review. J Pak Med Assoc.

[51] Fatima A, Shafique MA, Alam K, Fadlalla Ahmed TK, Mustafa MS (2024). ChatGPT in medicine: a cross-disciplinary systematic review of ChatGPT’s (artificial intelligence) role in research, clinical practice, education, and patient interaction. Medicine (Baltimore).

[52] Rehman M, Shafi I, Ahmad J, Garcia CO, Barrera AEP, Ashraf I (2025). Advancement in medical report generation: current practices, challenges, and future directions. Med Biol Eng Comput.

[53] Fareed M, Fatima M, Uddin J, Ahmed A, Sattar MA (2025). A systematic review of ethical considerations of large language models in healthcare and medicine. Front Digit Health.

[54] Nasra M, Jaffri R, Pavlin-Premrl D (2025). Can artificial intelligence improve patient educational material readability? A systematic review and narrative synthesis. Intern Med J.

[55] Sanjeewa R, Iyer R, Apputhurai P, Wickramasinghe N, Meyer D (2024). Empathic conversational agent platform designs and their evaluation in the context of mental health: systematic review. JMIR Ment Health.

[56] Omar M, Soffer S, Charney AW, Landi I, Nadkarni GN, Klang E (2024). Applications of large language models in psychiatry: a systematic review. Front Psychiatry.

[57] Omar M, Levkovich I (2025). Exploring the efficacy and potential of large language models for depression: a systematic review. J Affect Disord.

[58] Pashangpour S, Nejat G (2024). The future of intelligent healthcare: a systematic analysis and discussion on the integration and impact of robots using large language models for healthcare. Robotics.

[59] Sacoransky E, Kwan BYM, Soboleski D (2024). ChatGPT and assistive AI in structured radiology reporting: a systematic review. Curr Probl Diagn Radiol.

[60] Bečulić H, Begagić E, Skomorac R, Mašović A, Selimović E, Pojskić M (2024). ChatGPT’s contributions to the evolution of neurosurgical practice and education: a systematic review of benefits, concerns and limitations. Med Glas (Zenica).

[61] Garg RK, Urs VL, Agarwal AA, Chaudhary SK, Paliwal V, Kar SK (2023). Exploring the role of ChatGPT in patient care (diagnosis and treatment) and medical research: a systematic review. Health Promot Perspect.

[62] Haltaufderheide J, Ranisch R (2024). The ethics of ChatGPT in medicine and healthcare: a systematic review on large language models (LLMs). NPJ Digit Med.

[63] Kiuchi K, Otsu K, Hayashi Y (2024). Psychological insights into the research and practice of embodied conversational agents, chatbots and social assistive robots: a systematic meta-review. Behav Inf Technol.

[64] Kolding S, Lundin RM, Hansen L, Østergaard SD (2024). Use of generative artificial intelligence (AI) in psychiatry and mental health care: a systematic review. Acta Neuropsychiatr.

[65] Mohamed YA, Mohamed AHHM, Khanan A, Bashir M, Adiel MAE, Elsadig MA (2024). Navigating the ethical terrain of AI-generated text tools: a review. IEEE Access.

[66] Paganelli A, Spadafora M, Navarrete-Dechent C, Guida S, Pellacani G, Longo C (2024). Natural language processing in dermatology: a systematic literature review and state of the art. J Eur Acad Dermatol Venereol.

[67] Kutbi M (2024). Artificial intelligence-based applications for bone fracture detection using medical images: a systematic review. Diagnostics (Basel).

[68] Roman A, Al-Sharif L, AL Gharyani M (2023). The expanding role of ChatGPT (Chat-Generative Pre-Trained Transformer) in neurosurgery: a systematic review of literature and conceptual framework. Cureus.

[69] Rudnicka Z, Proniewska K, Perkins M, Pregowska A (2024). Cardiac healthcare digital twins supported by artificial intelligence-based algorithms and extended reality—a systematic review. Electronics (Basel).

[70] Ruksakulpiwat S, Kumar A, Ajibade A (2023). Using ChatGPT in medical research: current status and future directions. J Multidiscip Healthc.

[71] Moya-Salazar J, Salazar CR, Delzo SS, Goicochea-Palomino EA, Rojas-Zumaran V (2024). After a few months, what are the uses of OpenAI’s ChatGPT in medicine? A Scopus-based systematic review. Electron J Gen Med.

[72] Sallam M (2023). ChatGPT utility in healthcare education, research, and practice: systematic review on the promising perspectives and valid concerns. Healthcare (Basel).

[73] Banskota B, Bhusal R, Yadav PK, Banskota AK (2025). Artificial intelligence in orthopaedic education, training and research: a systematic review. BMC Med Educ.

[74] McHugh ML (2012). Interrater reliability: the kappa statistic. Biochem Med (Zagreb).

[75] Facts and figures. Genetic Alliance UK.

[76] Wang L, Wan Z, Ni C (2024). A systematic review of chatgpt and other conversational large language models in healthcare. medRxiv.

[77] (2024). Confronting antimicrobial resistance 2024 to 2029. GOVUK.

[78] Katirai A (2024). The environmental costs of artificial intelligence for healthcare. ABR.

[79] Cowls J, Tsamados A, Taddeo M, Floridi L (2023). The AI gambit: leveraging artificial intelligence to combat climate change-opportunities, challenges, and recommendations. AI Soc.

[80] Bai X, Wang A, Sucholutsky I, Griffiths TL (2025). Explicitly unbiased large language models still form biased associations. Proc Natl Acad Sci U S A.

[81] Hatem R, Simmons B, Thornton JE (2023). A call to address AI “hallucinations” and how healthcare professionals can mitigate their risks. Cureus.

[82] Thaler SL (1995). “Virtual input” phenomena within the death of a simple pattern associator. Neural Netw.

[83] Maleki N, Padmanabhan B, Dutta K (2024). 2024 IEEE Conference on Artificial Intelligence (CAI.

[84] Boksa P (2009). On the neurobiology of hallucinations. J Psychiatry Neurosci.

[85] Wu WT, Chao YW, Lin TK, Huang CK, Hsieh PH (2025). Economic evaluation of AI-assisted technologies in healthcare: a systematic review. J Food Drug Anal.

