# Supplementary material for: Concerns of Using Large Language Models in Health Care Research and Practice: Umbrella Review
Source: J Med Internet Res. 2026 May 15;28:e87804. doi: 10.2196/87804 (PMC13181733; doi:10.2196/87804)
Supplement: Multimedia Appendix 1 [file jmir-v28-e87804-s001.docx]

**Medline (Ovid)**

((ethic* or concern* or raises questions or equality or equity or racial or discriminat* or EDI or (equity diversity and inclusion) or adversely or perpetuat* or persist* or bolster or pitfall* or controvers* or worry or barrier or impede or obstacle or limitation or hinderance or hurdle) and ((LLM or large language model or GenAI or generative AI or ChatGPT or openAI or gpt or Gemini or DeepSeek or LlaMA or Falcon or Cohere or PaLM or Claude v1 or autoregressive language or encoder-decoder or decoder or transformer or prompt engineer) and (research or academ*))).ti,ab,kw.

Limitations: date, 2017 onwards

Filters: Systematic review and Review (Maximise specificity)

Update: limit to dT=”20250225-20260225”

**Embase (Ovid)**

((ethic* or concern* or raises questions or equality or equity or racial or discriminat* or EDI or (equity diversity and inclusion) or adversely or perpetuat* or persist* or bolster or pitfall* or controvers* or worry or barrier or impede or obstacle or limitation or hinderance or hurdle) and ((LLM or large language model or GenAI or generative AI or ChatGPT or openAI or gpt or Gemini or DeepSeek or LlaMA or Falcon or Cohere or PaLM or Claude v1 or autoregressive language or encoder-decoder or decoder or transformer or prompt engineer) and (research or academ*))).ti,ab,kw.

Limitations: date, 2017 onwards

Filters: Review and Review (Maximise specificity)

Update: limit to dc=”20250225-20260225”

**Scopus**

TITLE-ABS-KEY ( ( ( ethic* OR concern* OR raises questions OR equality OR equity OR racial OR discriminat* OR EDI OR ( equity diversity and inclusion ) OR adversely OR perpetuat* OR persist* OR bolster OR pitfall* OR controvers* OR worry OR barrier OR impede OR obstacle OR limitation OR hinderance OR hurdle ) AND ( ( LLM OR large LANGUAGE model OR GenAI OR generative AI OR ChatGPT OR openAI OR gpt OR Gemini OR DeepSeek OR LlaMA OR Falcon OR Cohere OR PaLM OR Claude v1 OR autoregressive LANGUAGE OR encoder-decoder OR decoder OR transformer OR prompt engineer ) AND ( research OR academ* ) ) ) )

Limitations: date, 2017 onwards

Filters: None

Update: limit date, 2025 onwards

**Web of Science**

(((((((((((((((((((((((TS = ethic*) OR (TS = concern*)) OR (TS = raises questions)) OR (TS = equality)) OR (TS = equity)) OR (TS = racial)) OR (TS = discriminat*)) OR (TS = EDI)) OR ((TS = equity diversity) AND (TS = inclusion))) OR (TS = adversely)) OR (TS = perpetuat*)) OR (TS = persist*)) OR (TS = bolster)) OR (TS = pitfall*)) OR (TS = controvers*)) OR (TS = worry)) OR (TS = barrier)) OR (TS = impede)) OR (TS = obstacle)) OR (TS = limitation)) OR (TS = hinderance)) OR (TS = hurdle)) AND ((((((((((((((((((((TS = LLM) OR (TS = large LANGUAGE model)) OR (TS = GenAI)) OR (TS = generative AI)) OR (TS = ChatGPT)) OR (TS = openAI)) OR (TS = gpt)) OR (TS = Gemini)) OR (TS = DeepSeek)) OR (TS = LlaMA)) OR (TS = Falcon)) OR (TS = Cohere)) OR (TS = PaLM)) OR (TS = claude v1)) OR (TS = autoregressive language)) OR (TS = encoder-decoder)) OR (TS = decoder)) OR (TS = transformer)) OR (TS = prompt engineer)) AND ((TS = research) OR (TS = academ*))))

Limitations: date, 2017 onwards

Filters: Review article

Update: limit date 25-02-2025 to 25-02-2026

**JBI Database of Systematic Reviews and Implementation Reports**

( ( ( ethical OR ethics OR concern* OR concerns OR concerned OR raises questions OR equality OR equity OR racial OR discrimination OR discriminatory OR EDI OR ( equity AND diversity AND inclusion ) OR adversely OR perpetuates OR perpetuated OR persists OR persistent OR persisted OR bolster OR pitfall OR pitfalls OR controversial OR controversy OR worry OR barrier OR impede OR obstacle OR limitation OR hinderance OR hurdle ) AND ( ( LLM OR large LANGUAGE model OR GenAI OR generative AI OR ChatGPT OR openAI OR gpt OR Gemini OR DeepSeek OR LlaMA OR Falcon OR Cohere OR PaLM OR Claude v1 OR autoregressive LANGUAGE OR encoder-decoder OR decoder OR transformer OR prompt engineer ) AND ( research OR academic OR academia ) ) ) )

Limitations: date, 2017 onwards

Filters: None

Update: limit date to 2025 onwards

**Cochrane database of systematic reviews**

(((ethic* or concern* or raises questions or equality or equity or racial or discriminat* or EDI or (equity diversity and inclusion) or adversely or perpetuat* or persist* or bolster or pitfall* or controvers* or worry or barrier or impede or obstacle or limitation or hinderance or hurdle) and ((LLM or large language model or GenAI or generative AI or ChatGPT or openAI or gpt or Gemini or DeepSeek or LlaMA or Falcon or Cohere or PaLM or Claude v1 or autoregressive language or encoder-decoder or decoder or transformer or prompt engineer) and (research or academ*)))):ti,ab,kw

Limitations: date, 2017 onwards

Filters: None

Update: limit date, Feb 2025 onwards

**Epistemonikos**

(title:((((ethic* OR concern* OR raises questions OR equality OR equity OR racial OR discriminat* OR EDI OR (equity diversity AND inclusion) OR adversely OR perpetuat* OR persist* OR bolster OR pitfall* OR controvers* OR worry OR barrier OR impede OR obstacle OR limitation OR hinderance OR hurdle) AND ((LLM OR large language model OR GenAI OR generative AI OR ChatGPT OR openAI OR gpt OR Gemini OR DeepSeek OR LlaMA OR Falcon OR Cohere OR PaLM OR Claude v1 OR autoregressive language OR encoder-decoder OR decoder OR transformer OR prompt engineer) AND (research OR academ*))))) OR abstract:((((ethic* OR concern* OR raises questions OR equality OR equity OR racial OR discriminat* OR EDI OR (equity diversity AND inclusion) OR adversely OR perpetuat* OR persist* OR bolster OR pitfall* OR controvers* OR worry OR barrier OR impede OR obstacle OR limitation OR hinderance OR hurdle) AND ((LLM OR large language model OR GenAI OR generative AI OR ChatGPT OR openAI OR gpt OR Gemini OR DeepSeek OR LlaMA OR Falcon OR Cohere OR PaLM OR Claude v1 OR autoregressive language OR encoder-decoder OR decoder OR transformer OR prompt engineer) AND (research OR academ*))))))

Limitations: date, 2017 onwards

Filters: Systematic review; Systematic Review Question = Qualitative

Update: limit date, 2025 onwards
