# Supplementary material for: Concerns of Using Large Language Models in Health Care Research and Practice: Umbrella Review
Source: J Med Internet Res. 2026 May 15;28:e87804. doi: 10.2196/87804 (PMC13181733; doi:10.2196/87804)
Supplement: Multimedia Appendix 2 [file jmir-v28-e87804-s002.docx]

**List of excluded articles**

Detailed below are the full references for all studies excluded at full text stage. Articles were excluded for one of the following reasons:

1. Wrong outcomes/concerns not assessed (n = 13)
2. Not a systematic review (n = 10)
3. Not about health or social care (n = 2)
4. Paywalled article (n = 2)

| **Reason for exclusion** | **References** |
| --- | --- |
| Wrong outcomes/concerns not assessed (n = 12) | 1. Ali, D., Fatemi, Y., Boskabadi, E., Nikfar, M., Ugwuoke, J., & Ali, H. (2024). ChatGPT in Teaching and Learning: A Systematic Review. EDUCATION SCIENCES, 14(6). https://doi.org/10.3390/educsci14060643 2. Bacco, L., Russo, F., Ambrosio, L., D'Antoni, F., Vollero, L., Vadalà, G., Dell'Orletta, F., Merone, M., Papalia, R., & Denaro, V. (2022). Natural language processing in low back pain and spine diseases: A systematic review. FRONTIERS IN SURGERY, 9. 3. Bouhafra, S., & El Bahi, H. (2024). Deep Learning Approaches for Brain Tumor Detection and Classification Using MRI Images (2020 to 2024): A Systematic Review. JOURNAL OF IMAGING INFORMATICS IN MEDICINE. <https://doi.org/10.1007/s10278-024-01283-8> 4. Deng, J., & Qin, Y. (2024). Current Status, Hotspots, and Prospects of Artificial Intelligence in Ophthalmology: A Bibliometric Analysis (2003-2023). Ophthalmic Epidemiology. https://doi.org/https://dx.doi.org/10.1080/09286586.2024.2373956 PT - Article In Press 5. Gunawan, J., Aungsuroch, Y., & Montayre, J. (2024). ChatGPT integration within nursing education and its implications for nursing students: A systematic review and text network analysis. Nurse education today, 141, 106323. https://doi.org/https://dx.doi.org/10.1016/j.nedt.2024.106323 PT - Review 6. Liu, Y., Wang, H., Zhou, H., Li, M., Hou, Y., Zhou, S., Wang, F., Hoetzlein, R., & Zhang, R. (2024). A review of reinforcement learning for natural language processing and applications in healthcare. Journal of the American Medical Informatics Association, 31(10), 2379 EP - 2393. https://doi.org/https://dx.doi.org/10.1093/jamia/ocae215 PT - Review 7. Oliveira, J. A., Eskandar, K., Kar, E., de Oliveira, F. R., & da Silva, A. L. (2024). Understanding AI's Role in Endometriosis Patient Education and Evaluating Its Information and Accuracy: Systematic Review. JMIR AI, 3. https://doi.org/10.2196/64593 8. Schopow, N., Osterhoff, G., & Baur, D. (2023). Applications of the Natural Language Processing Tool ChatGPT in Clinical Practice: Comparative Study and Augmented Systematic Review. JMIR MEDICAL INFORMATICS, 11. https://doi.org/10.2196/48933 9. Solano, C., Tarazona, N., Angarita, G. P., Medina, A. A., Ruiz, S., Pedroza, V. M., & Traxer, O. (2024). ChatGPT in Urology: Bridging Knowledge and Practice for Tomorrow's Healthcare, a Comprehensive Review. Journal of Endourology, 38(8), 763 EP - 777. https://doi.org/https://dx.doi.org/10.1089/end.2023.0700 PT - Review 10. Sorin, V., Brin, D., Barash, Y., Konen, E., Charney, A., Nadkarni, G., & Klang, E. (2024). Large Language Models and Empathy: Systematic Review. J Med Internet Res, 26, e52597. https://doi.org/10.2196/52597 11. Spasic, I., & Nenadic, G. (2020). Clinical Text Data in Machine Learning: Systematic Review. JMIR MEDICAL INFORMATICS, 8(3). <https://doi.org/10.2196/17984> 12. Zahidy, M. A. L., Montori, V., & Ponce, O. J. (2024). CO-CREATING THE FUTURE: AI FOR ASSESSING AND ENABLING SHARED DECISION MAKING. BMJ Evidence-Based Medicine, 29, A2. https://doi.org/https://dx.doi.org/10.1136/bmjebm-2024-SDC.5 PT - Conference Abstract (12th International Shared Decision Making Conference. Lausanne Switzerland.) 13. Zhou, S., et al. (2025). "Mitigating Ethical Issues for Large Language Models in Oncology: A Systematic Review." JCO Clinical Cancer Informatics(9): e2500076. |
| Not a systematic review (n = 10) | 1. Cheng, S. W., Chang, C. W., Chang, W. J., Wang, H. W., Liang, C. S., Kishimoto, T., Chang, J. P. C., Kuo, J. S., & Su, K. P. (2023). The now and future of ChatGPT and GPT in psychiatry. PSYCHIATRY AND CLINICAL NEUROSCIENCES, 77(11), 592-596. <https://doi.org/10.1111/pcn.13588> 2. Giunti, G., & Doherty, C. P. (2024). Cocreating an Automated mHealth Apps Systematic Review Process With Generative AI: Design Science Research Approach. JMIR MEDICAL EDUCATION, 10. <https://doi.org/10.2196/48949> 3. Ihara, K., Dumkrieger, G., Zhang, P., Takizawa, T., Schwedt, T. J., & Chiang, C. C. (2024). Application of Artificial Intelligence in the Headache Field. Current Pain and Headache Reports. https://doi.org/https://dx.doi.org/10.1007/s11916-024-01297-5 PT - Article In Press 4. Lim, D. Y., Tan, Y. B., Koh, J., Sng, G., Tung, J., Le, Q., Tan, J. H., Tan, D. M. Y., Ting, D., & Tan, C. K. (2024). A SYSTEMATIC REVIEW OF LARGE LEARNING MODELS IN GASTROENTEROLOGY AND HEPATOLOGY - APPLICATIONS AND TECHNIQUES TO MITIGATE MISINFORMATION. Gastroenterology, 166(5), S EP - 1489. https://doi.org/https://dx.doi.org/10.1016/S0016-5085%2824%2903862-9 PT - Conference Abstract (DDW 2024. Washington, DC United States.) 5. Neubauer, L., Straw, I., Mariconti, E., & Tanczer, L. M. (2023). A Systematic Literature Review of the Use of Computational Text Analysis Methods in Intimate Partner Violence Research. JOURNAL OF FAMILY VIOLENCE, 38(6), 1205-1224. <https://doi.org/10.1007/s10896-023-00517-7> 6. Owen, D., Lynham, A. J., Smart, S. E., Pardiñas, A. F., & Collados, J. C. (2024). AI for Analyzing Mental Health Disorders Among Social Media Users: Quarter-Century Narrative Review of Progress and Challenges. Journal of Medical Internet Research, 26. <https://doi.org/10.2196/59225> 7. Tei, S., & Fujino, J. (2024). Artificial intelligence, Internet addiction, and palliative care. European Psychiatry, 67, S339. https://doi.org/https://dx.doi.org/10.1192/j.eurpsy.2024.700 PT - Conference Abstract (32nd European Congress of Psychiatry, EPA 2024. Budapest Hungary.) 8. Theodosiou, A. A., & Read, R. C. (2023). Artificial intelligence, machine learning and deep learning: Potential resources for the infection clinician. JOURNAL OF INFECTION, 87(4), 287-294. https://doi.org/10.1016/j.jinf.2023.07.006 9. Yeskuatov, E., Chua, S. L., & Foo, L. K. (2022). Leveraging Reddit for Suicidal Ideation Detection: A Review of Machine Learning and Natural Language Processing Techniques. INTERNATIONAL JOURNAL OF ENVIRONMENTAL RESEARCH AND PUBLIC HEALTH, 19(16). <https://doi.org/10.3390/ijerph191610347>   Zhang, H., Xi, Q. Y., Zhang, F., Li, Q. X., Jiao, Z. Q., & Ni, X. Y. (2023). Application of Deep Learning in Cancer Prognosis Prediction Model. TECHNOLOGY IN CANCER RESEARCH & TREATMENT, 22. https://doi.org/10.1177/15330338231199287 |
| Not about health or social care (n = 2) | 1. Al-kfairy, M., Mustafa, D., Kshetri, N., Insiew, M., & Alfandi, O. (2024). Ethical Challenges and Solutions of Generative AI: An Interdisciplinary Perspective. INFORMATICS-BASEL, 11(3). <https://doi.org/10.3390/informatics11030058> 2. Bavalatti, T., Ahmed, O., Potdar, D., Rouf, R., Jawed, F., Govind, M. K., & Krishnan, S. (2025). A Systematic Review of Open Datasets used in Text-to-Image (T2I) Gen AI Model Safety. IEEE ACCESS. [https://doi.org/10.1109/ACCESS.2025.3539933#](https://doi.org/10.1109/ACCESS.2025.3539933) |
| Paywalled article (n = 2) | 1. Kalaw, F. G. P., & Baxter, S. L. (2024). Ethical considerations for large language models in ophthalmology. Current Opinion in Ophthalmology, 35(6), 438 EP - 446. https://doi.org/https://dx.doi.org/10.1097/ICU.0000000000001083 PT - Review 2. Solano, C., Tarazona, N., Angarita, G. P., Medina, A. A., Ruiz, S., Pedroza, V. M., & Traxer, O. (2024). ChatGPT in Urology: Bridging Knowledge and Practice for Tomorrow's Healthcare, a Comprehensive Review. Journal of Endourology, 38(8), 763 EP - 777. https://doi.org/https://dx.doi.org/10.1089/end.2023.0700 PT - Review |
